# Polyherbal therapy modulates survival, hematological parameters, and gill health in *Aeromonas veronii*-infected *Cyprinus carpio*

**DOI:** 10.3389/fvets.2026.1889901

**Published:** 2026-08-19

**Authors:** Omed I. Abid, Nasreen M. Abdulrahman

**Affiliations:** 1 Sulaimani Veterinary Laboratory, Sulaimani Directorate, Sulaymaniyah, Iraq; 2 Masi Altuni Company, Sulaymaniyah, Iraq; 3 College of Veterinary Medicine, University of Sulaimani, Sulaymaniyah, Iraq

**Keywords:** *Aeromonas veronii*, aquaculture, *Cyprinus carpio*, fish disease, herbal medicine, polyherbal therapy

## Abstract

This study evaluated the protective effects of pomegranate peel, garlic, and a polyherbal mixture (PHM) against *Aeromonas veronii*-associated gill disease in common carp (*Cyprinus carpio*). A total of 234 healthy fish were allocated into 13 groups, including one uninfected control group and 12 experimentally infected groups. Infected fish received diets supplemented with oxytetracycline, garlic (2.5%, 5%, and 7.5%), pomegranate peel (2.5%, 5%, and 7.5%), or PHM at equivalent concentrations for 14 days. Untreated infected fish and garlic-treated groups exhibited 100% mortality within 14 days post-infection. In contrast, PHM supplementation significantly improved survival, achieving up to 94% survival (*P* < 0.05). PHM-treated fish also demonstrated improved hematological parameters, enhanced immune-associated responses, and reduced alterations in liver enzyme activities compared with infected controls. Histopathological examination revealed that PHM preserved gill architecture and reduced epithelial degeneration, necrosis, and hyperplasia associated with *A. veronii* infection. These findings demonstrate that dietary PHM supplementation enhances resistance to *A. veronii* infection and improves several health-related parameters in experimentally challenged common carp. The observed protective effects may be associated with the bioactive phytochemical constituents identified by GC–MS analysis; however, the precise mechanisms of action remain to be elucidated. Furthermore, additional studies are required to evaluate long-term safety under non-challenged conditions and to further characterize the biological activities of PHM.

## 1 Introduction

Today, herbal medicines are increasingly being investigated as sustainable alternatives to conventional antibiotics in aquaculture because of their broad spectrum of biological activities and lower environmental impact. For instance, pomegranate (*Punica granatum*) peel is a rich source of flavonoids, phenolic acids, and tannins with documented antimicrobial and antioxidant properties. Previous studies have demonstrated that pomegranate peel supplementation can enhance disease resistance and reduce bacterial infections in fish species (1). Rosemary (*Rosmarinus officinalis*) possesses antibacterial activity against both Gram-positive and Gram-negative bacteria and has been reported to improve antioxidant status, immune-associated responses, and stress tolerance in common carp (*Cyprinus carpio)* (2). Similarly, black cumin (*Nigella sativa*) and its oil exhibit antimicrobial activity and have been associated with improved survival and disease resistance in cultured fish under intensive production conditions (3). Fenugreek (*Trigonella foenum-graecum*) contains numerous bioactive compounds with anti-inflammatory and antimicrobial activities and has been shown to enhance growth performance, antioxidant capacity, and immune-associated responses in fish (4). Sesamol, a phenolic compound derived from sesame (*Sesamum indicum*), possesses antimicrobial, antioxidant, immunomodulatory, and cytoprotective properties (5). Peppermint (*Mentha piperita*) contains menthol and other bioactive constituents that support digestive function and may improve nutrient utilization and gastrointestinal health (6). Thyme (*Thymus vulgaris*) contains thymol, carvacrol, cymene, eugenol, and related phytochemicals that have demonstrated antimicrobial, antioxidant, growth-promoting, and immune-supportive activities in aquaculture species (7).

Furthermore, previous studies have reported beneficial effects of individual herbal supplements such as garlic, pomegranate peel, black cumin, and thyme against bacterial infections in fish. However, the efficacy of single-herb interventions can vary considerably depending on pathogen species, strain characteristics, dosage, preparation methods, and environmental conditions. For example, although garlic has demonstrated antibacterial activity against several aquatic pathogens, its effectiveness against different *Aeromonas* species has been inconsistent across studies (8, 9). Consequently, combining medicinal plants with complementary biological properties may provide broader biological activity than single-herb approaches by simultaneously targeting multiple physiological and pathological processes associated with infection.

The effectiveness of polyherbal formulations in aquaculture has attracted increasing research attention (10–13). Several studies have demonstrated that multi-herb dietary supplements can improve growth performance, stimulate digestive enzyme activity, enhance antioxidant defenses, and strengthen immune-associated responses in cultured fish. For example, polyherbal supplementation improved growth, digestive function, and physiological performance in European eel (*Anguilla anguilla*) (11) and juvenile Japanese seabass (*Lateolabrax japonicus*) (13), while a formulation containing *Coriandrum sativum, Malva sylvestris*, and *Quercus brantii* enhanced non-specific immunity, growth performance, and resistance to *Aeromonas hydrophila* infection in common carp (12). Despite these promising findings, information remains limited regarding the effects of polyherbal formulations on *Aeromonas veronii*-associated disease in common carp. Furthermore, the potential benefits of combining multiple medicinal plants with complementary antimicrobial, antioxidant, digestive-supportive, and immune-associated properties have not been adequately investigated in this infection model.

Therefore, the present study evaluated the effects of garlic, pomegranate peel, and a polyherbal mixture (PHM) consisting of black cumin, pomegranate peel, rosemary, fenugreek, sesame, peppermint, and thyme in common carp experimentally challenged with *A. veronii*. The formulation was developed based on published evidence supporting the biological activities of its individual components rather than experimentally validated synergistic interactions (14–17). Particular emphasis was placed on survival rate, hematological parameters, immune-associated responses, liver enzyme activities, and gill histopathological alterations.

## 2 Materials and methods

### 2.1 Preparation and chemical analysis of medicinal plants

Dried garlic (*Allium sativum*), black cumin (*Nigella sativa*), pomegranate peel (*Punica granatum*), rosemary (*Rosmarinus officinalis*), fenugreek (*Trigonella foenum-graecum*), sesame (*Sesamum indicum*), peppermint (*Mentha piperita*), and thyme (*Thymus vulgaris*) were obtained from a local supplier in Sulaymaniyah, Iraq. A polyherbal mixture (PHM) was formulated in a ratio of 2:2:1:1:1:1:1 consisting of black cumin, pomegranate peel, rosemary, fenugreek, sesame, peppermint, and thyme, respectively. The formulation was developed based on published evidence demonstrating the antimicrobial, antioxidant, and immune-associated properties of these medicinal plants. Black cumin and pomegranate peel were included at higher proportions because previous studies have reported their potent antibacterial activity, antioxidant capacity, and ability to enhance immune responses and disease resistance in fish and other animal species (1, 3). Rosemary, fenugreek, sesame, peppermint, and thyme were incorporated at equal proportions to provide complementary antioxidant, anti-inflammatory, digestive, and immune-supportive effects (2, 4–7). Therefore, the selected formulation was based on literature-supported functional properties of the individual components rather than experimentally validated synergy testing. All plant materials were ground into a fine powder using a laboratory mill and stored in airtight containers until use.

Proximate chemical analyses of garlic powder, pomegranate peel powder, and PHM were conducted according to the standard procedures of the Association of Official Analytical Chemists (18). The analyzed parameters included crude protein, ether extract, crude fiber, dry matter, moisture, and ash content. Nitrogen-free extract (NFE) was calculated by subtracting the percentages of protein, fat, moisture, ash, and fiber from 100%. In addition, the phytochemical composition of garlic, pomegranate peel, and PHM extracts was characterized using gas chromatography–mass spectrometry (GC–MS) to identify major bioactive constituents potentially associated with their biological activities. However, quantitative determinations of total phenolic content (TPC), total flavonoid content (TFC), tannins, and antioxidant capacity (e.g., DPPH, ABTS, and FRAP assays) were beyond the scope of the present study and were not evaluated.

### 2.2 Fish husbandry, experimental challenge, and experimental design

A total of 234 apparently healthy common carp (*Cyprinus carpio*) with an average weight of 32 ± 7 g and body length of 13 ± 2 cm were obtained from a local hatchery. Fish health status was assessed according to EPA guidelines (19). Fish were acclimatized for 12 days in aerated fiberglass tanks containing 60 L of dechlorinated water under controlled conditions. Water quality was maintained at a temperature of 27.5 ± 0.8 °C, pH 7.5 ± 0.8, and dissolved oxygen levels of ≥4.7 mg/L. These physicochemical parameters were monitored using a MesuLab M510T multi-parameter analyzer. To preserve optimal water quality, approximately 80% of the tank water was replaced daily, allowing for the removal of uneaten feed and accumulated waste (Supplementary Table S1).

Twelve experimental groups were challenged by immersion exposure to *A. veronii*. Each tank containing 18 fish received 6 mL of bacterial suspension (3 × 10^8^ CFU/mL) for 24 h, resulting in a final concentration of approximately 3 × 10^7^ CFU/mL under continuous aeration. One additional group served as the non-infected control and was exposed to sterile distilled water only.

Following bacterial exposure, each treatment group was divided into three replicate tanks containing six fish per tank. Each replicate tank was maintained independently and considered the experimental unit for statistical analyses. Fish were randomly assigned to treatment groups and replicate tanks to minimize allocation bias. The replication structure was selected based on previously published aquaculture challenge studies employing comparable bacterial infection models and logistical considerations associated with maintaining multiple treatment groups under controlled laboratory conditions (20). However, an *a priori* statistical power analysis was not performed, and this should be considered a limitation when interpreting treatment effects.

Experimental fish were fed once daily at 4% of body weight according to the designated dietary treatments for 14 days. Experimental groups included oxytetracycline-treated fish, garlic-supplemented diets, pomegranate peel-supplemented diets, and PHM-supplemented diets at concentrations of 2.5%, 5%, and 7.5% (Tables 1, 2). Fish were monitored daily for behavioral changes, clinical signs, feed intake, and mortality throughout the experimental period.

**Table 1:** Experimental groups were randomly designed in three replicated six fish each.

Experimental groups	Treatments for Aeromonas veronii-Infected Fish
T1	Control group
T2	Infected with *A. veronii*
T3	Infected with *A. veronii* and administered Oxytetracycline 75 mg/kg of feed
T4	Infected with *A. veronii* and administered Oxytetracycline 10 mg/L daily
T5	Infected with *A. veronii* and administered a feed contains 2.5% of garlic powder
T6	Infected with *A. veronii* and administered a feed contains 5% of garlic powder
T7	Infected with *A. veronii* and administered a feed contains 7.5% of garlic powder
T8	Infected with *A. veronii* and administered a feed contains 2.5% of pomegranate peel powder
T9	Infected with *A. veronii* and administered a feed contains 5% of pomegranate peel powder
T10	Infected with *A. veronii* and administered a feed contains 7.5% of pomegranate peel powder
T11	Infected with *A. veronii* and administered a feed contains 2.5% of poly-herbal mixture powder
T12	Infected with *A. veronii* and administered a feed contains 5% of poly-herbal mixture powder
T13	Infected with *A. veronii* and administered a feed contains 7.5% of poly-herbal mixture powder

**Table 2:** Experimental diet fed by fish during 14 days of the experiment.

Ingredients	Control	Garlic			Poy-herbal Mixture			Pomegranate		
		**2.5%**	**5%**	**7.5%**	**2.5%**	**5%**	**7.5%**	**2.5%**	**5%**	**7.5%**
Corn grain	37.633	34.554	31.239	30.3	36.236	34.601	33.602	34.554	31.239	30.239
Soybean meal 46%	54.219	53.832	54.060	53	53.014	52.425	51.424	53.832	54.060	53.060
Sunflower oil	2.498	2.935	3.511	3.01	2.063	1.767	1.267	2.935	3.511	3.011
Dicalcium-phosphate	1.690	1.713	1.726	1.726	1.715	1.732	1.732	1.713	1.726	1.726
CaCO_3_	1.219	1.210	1.202	1.202	1.214	1.209	1.209	1.210	1.202	1.202
Common salt	0.133	0.186	0.188	0.188	0.186	0.187	0.188	0.186	0.188	0.188
Concentrate^a^	2.500	2.500	2.500	2.5	2.500	2.500	2.500	2.500	2.500	2.500
Methionine	0.108	0.570	0.574	0.574	0.572	0.579	0.578	0.570	0.574	0.574
Garlic	0.000	2.500	5.000	7.500	0.000	0.000	0.000	0.000	0.000	0.000
Poy-herbal mixture	0.000	0.000	0.000	0.000	2.500	5.000	7.500	0.000	0.000	0.000
Pomegranate	0.000	0.000	0.000	0.000	0.000	0.000	0.000	2.500	5.000	7.500
**Chemical composition**			
M.Energy Kcal/kg	2,975	2,975	2,975	2,975	2,975	2,975	2,975	2,975	2,975	2,975
Crude protein%	29.000	29.000	29.000	29.000	29.000	29.000	29.000	29.000	29.000	29.000
Ether extract%	4.594	4.954	5.456	4.661	4.671	4.891	4.954	4.954	5.456	4.954
Linoleic acid%	2.319	2.473	2.694	2.071	2.061	1.872	2.473	2.473	2.694	2.473
Crude fiber%	2.816	2.744	2.674	3.110	3.110	3.406	3.269	3.269	3.724	3.269
Calcium%	1.000	1.000	1.000	1.000	1.000	1.000	1.000	1.000	1.000	1.000
Available phosphate%	0.500	0.500	0.500	0.500	0.500	0.500	0.500	0.500	0.500	0.500
Lysine%	1.656	1.638	1.637	1.619	1.619	1.599	1.638	1.638	1.637	1.638
TSAA%	0.909	1.351	1.347	1.349	1.349	1.342	1.00	1.00	1.347	1.00

M.Energy, metabolizable energy; TSAA, total sulfur amino acids.

a Provided per kg of diet: Crude ash, 72%; Crude fiber, 0.80%; Crude fat, 0.30%; Protein, 20.30%; Calcium (carbonate), 14.00%; Phosphorus (MCP),4.55%; Magnesium, 0.30%; Sodium chloride, 6.50%; Methionine, 8.30%; Lysine, 7.40%; Threonine, 2.70%; Tryptophane; 0.40%; Iron (Sulfate), 2,400 mg; Zinc (Oxide), 2,500 mg; Manganese (Oxide), 3,300 mg; Copper (Sulfate), 600 mg; iodine (IK), 85 mg; Selenium (SeNa),12 mg; Cobalt (Sulfate),2 mg; Phytase, 45,000 FYT; b-Glucanase,2,800 IU; b-Xylanase, 11,000 IU; Vitamin A, 400,000 IU; Vitamin D3. 120,000 IU; Vitamin E, 2,000 mg; VitaminK3, 100 mg; Vitamin B1, 130 mg; Vitamin B2, 320 mg; Vitamin B6, 210 mg; Vitamin B12, 1.2 mcg; Biotine, 6,000 mg; Folic acid, 60 mg; Nicotinic acid, 2,000 mg; Pantothenic acid,600 mg; Choline, 20,000 mg; Citric acid, 60 mg; Colloidal silica, 120 mg; BHT, 200 mg; Sodium propionate,18 mg.

### 2.3 Hematological and biochemical analyses

Blood samples were collected from randomly selected fish on days 7 and 14 post-infection. Fish were anesthetized, and blood was collected from the caudal vein using sterile 2 mL syringes (21). Samples were transferred into EDTA-coated tubes for hematological analyses.

Hematological parameters, including total erythrocyte count (RBC), hemoglobin concentration (Hb), haematocrit (HCT), mean corpuscular volume (MCV), mean corpuscular hemoglobin (MCH), and mean corpuscular hemoglobin concentration (MCHC), were determined using standard hematological techniques. Cell counts were performed using a Neubauer haemocytometer and examined under a trinocular research microscope. Blood smears were stained with Giemsa stain and examined microscopically for differential leukocyte counts and cellular morphology according to Bardhan et al. (22).

For biochemical analyses, plasma was separated by centrifugation at 4,500 rpm for 15 min and stored at −20 °C until analysis. Alanine aminotransferase (ALT) and alkaline phosphatase (ALP) activities were measured using commercial diagnostic kits according to the manufacturers' instructions.

Blood and tissue sampling were conducted at 7 and 14 days post-infection (dpi) to evaluate intermediate and later-stage host responses to infection and treatment. Earlier sampling points were not included in the present study; therefore, acute-phase responses during the initial stages of infection were not assessed.

### 2.4 Histopathological examination of gill tissue

Gill samples were collected from fish on days 7 and 14 post-infection for histopathological evaluation. Tissue samples were fixed in 10% neutral buffered formalin, processed using standard histological procedures, embedded in paraffin wax, sectioned at 5 μm thickness, and stained with haematoxylin and eosin (H&E).

Histopathological alterations assessed included epithelial hyperplasia, chloride cell hyperplasia, mucus cell proliferation, epithelial lifting, lamellar fusion, oedema, vasodilatation, aneurysm formation, inflammatory infiltration, degeneration, and necrosis of gill filaments. Lesions were semi-quantitatively scored according to severity as follows: 0 = no detectable lesions; 1 = mild ( ≤ 15% tissue involvement); 2 = mild–moderate (16%−25%); 3 = moderate (26%−50%); 4 = moderate–severe (51%−75%); and 5 = severe (>75% tissue involvement) (23).

Histopathological slides were independently evaluated by two experienced pathologists who were blinded to treatment allocation and experimental grouping during lesion scoring. Representative photomicrographs illustrating the major histopathological alterations observed across treatment groups are provided in Supplementary Figures S1–S21.

### 2.5 Statistical analysis

Data were analyzed using the General Linear Model (GLM) procedure of SAS software version 9.1 (SAS Institute Inc., Cary, NC, USA) (24). Prior to statistical analysis, data were assessed for normality using the Shapiro–Wilk test and homogeneity of variance using Levene's test. Variables satisfying these assumptions were subjected to GLM analysis, followed by Tukey's *post hoc* multiple comparison test to identify differences among treatment groups.

Results are presented as mean ± standard error (SE). Statistical significance was accepted at *P* < 0.05. Letter-based groupings in tables indicate significant pairwise differences identified by Tukey's test. Exact *P*-values were reported where available. Mortality outcomes were summarized as cumulative mortality and survival percentages. Kaplan–Meier survival analysis and log-rank testing were not performed in the present study and should be considered in future investigations. Missing observations resulting from mortality were excluded from subsequent analyses because only surviving fish were available for sampling at each time point.

## 3 Results

### 3.1 Bioactive components of herbal extract

#### 3.1.1 Bioactive components of poly herbal mixture extract

Table 3 illustrates The GC–MS analysis revealed the presence of several phytochemical constituents with varying relative abundances and documented pharmacological activities. The compounds identified indicate that the sample is particularly rich in unsaturated fatty acids, with additional contributions from fatty acid esters, phenolic compounds, and terpenoids.

**Table 3:** Pharmacologically active constituents of a polyherbal mixture (PHM) ethanolic extract determined by GC-MS chromatography.

**Compounds**	Other name	RT	%	Pharmacological activity	Reference
Phenol, 2-methyl-5-(1-methylethyl)-	Carvacrol	14.092	0.22	Antimicrobial, antioxidant, and anticancer	Sharifi-Rad et al. (34)
Bicyclo[3.1.1]heptane, 2,6,6-trimethyl-, [1R-(1.alpha.,2.beta.,5.alpha.)]-	Pinane	22.691	0.08	Anti-inflammatory	Rufino et al. (35)
Dodecanoic acid, 10-methyl-, methyl ester	Methyl palmitate	23.968	0.08	Anti-inflammatory	Saeed et al. (36)
Hexadecanoic acid, ethyl ester	Palmitic acid, ethyl ester	24.627	11.93	Antibacterial	Yff et al. (37)
9,12-Octadecadienoic acid (Z,Z)-, methyl ester	Linoleic acid	24.903	38.5	Antibacterial	Dilika et al. (38)
11-Octadecenoic acid, methyl ester	Methyl 11-octadecenoate	25.368	0.34	Antibacterial	Ilozue et al. (39)
Oleic acid	Oleic acid	26.262	25.51	Antibacterial	Le et al. (40)
(E)-9-Octadecenoic acid ethyl ester	Elaidic acid	26.338	11.61	Antiviral	Andrei et al. (41)
Octadecanoic acid	Stearic acid	27.044	4.61	Antibacterial	Jubie et al. (42)
Octadecanoic acid, ethyl ester	Linoleic acid	27.097	7.12	Antimicrobial	Das (43)
SUM			100		

The most abundant compound identified was linoleic acid (9,12-octadecadienoic acid, (Z,Z)-), accounting for 38.50% of the total detected compounds (RT = 24.903 min). Linoleic acid is an essential polyunsaturated omega-6 fatty acid widely recognized for its antimicrobial, anti-inflammatory, antioxidant, and wound-healing properties. Its predominance suggests that it is the principal contributor to the biological activity of the extract, particularly its antibacterial potential.

The second most abundant compound was oleic acid, representing 25.51% of the total composition (RT = 26.262 min). Oleic acid is a monounsaturated omega-9 fatty acid that has been extensively reported to exhibit antibacterial, anti-inflammatory, antioxidant, and cardioprotective effects. The substantial concentration of oleic acid further supports the potential therapeutic value of the sample. Another major constituent was ethyl palmitate (hexadecanoic acid, ethyl ester), comprising 11.93% of the detected compounds (RT = 24.627 min). Ethyl palmitate has been reported to possess antibacterial activity and may also function as an emollient and antioxidant. Similarly, ethyl linoleate (9,12-octadecadienoic acid ethyl ester) accounted for 11.61% (RT = 26.338 min) and has been associated with antiviral, anti-inflammatory, and antioxidant properties.

Additional compounds detected include ethyl linoleate/octadecanoic acid ethyl ester (7.12%), stearic acid (octadecanoic acid) (4.61%), and methyl 11-octadecenoate (0.34%), all of which have been reported to exhibit antimicrobial or antibacterial activities. Although present in lower concentrations, these compounds may contribute synergistically to the overall biological activity of the extract.

Minor constituents identified were carvacrol (0.22%), pinane (bicyclo[3.1.1]heptane derivative) (0.08%), and methyl palmitate (0.08%). Despite their relatively low abundance, these compounds are pharmacologically significant. Carvacrol is a well-known phenolic monoterpenoid with potent antimicrobial, antioxidant, and anticancer properties, while pinane derivatives have demonstrated anti-inflammatory activity. Methyl palmitate has also been reported to possess anti-inflammatory effects.

#### 3.1.2 Bioactive components of garlic

Table 4 summarizes the GC–MS analysis of the bioactive constituents identified in the garlic extract, including their retention times (RT), relative percentages, and reported biological activities. The chromatographic profile demonstrated a complex chemical composition comprising furan derivatives, organosulfur compounds, fatty acids, phenolic compounds, terpenoids, amino acid derivatives, and vitamins. Many of these metabolites have been previously reported to possess antimicrobial, antioxidant, anti-inflammatory, and other pharmacological properties. The occurrence of these bioactive compounds provides scientific support for the traditional medicinal use of garlic and explains its diverse therapeutic potential.

**Table 4:** Pharmacologically active constituents of a garlic ethanolic extract determined by GC-MS chromatography.

Composition	Retention time	% of total	Pharmacological activity	References
Octadecanoic acid, 9,10-epoxy-, cis-	2.228	0.769	Antibacterial	Pu et al. (44)
Hexadecanoic acid	2.571	1	Antibacterial	Shaaban et al. (45)
l-Glutamic acid, monobenzyl ester	3.627	0.313	Antibacterial	Karimi et al. (46)
Furfural	3.947	6.980	Antibacterial	Chai et al. (47)
Dimethyl sulfoxide	5.074	2	Antibacterial	Kirkwood et al. (48)
Tetrahydropyran	5.871	1.042	Antibacterial	Akiyama et al. (49)
Methyl furfural	6.288	3.481	Antibacterial	Diaz-Morales et al. (50)
Methylmaleic anhydride	6.641	2.273	Antibacterial	Shaaban et al. (45)
β-Hydroxylauric acid	7.450	0.323	Antibacterial	Nakimera et al. (51)
n-Nonaldehyde	7.526	1.218	Antibacterial	Ramya et al. (52)
Melezitose	7.605	1	Antibacterial	Dżugan et al. (53)
Glycyl-D-threonine	8.012	1.759		Padmavathy et al. (54)
1H-Azonine, octahydro-1-nitroso-	8.396	4.409	Antioxidant	Fitriya (55)
Anethole	8.858	5	Antibacterial	Kubo et al. (56)
3-Hydroxy-2,3-dihydromaltol	9.266	7.896	Antibacterial	Meechai et al. (57)
2-Myristynoyl pantetheine	9.935	1.186	Antibacterial	Okechukwu et al. (58)
Nonanoic acid	10.184	1.458	Antibacterial	Sahin et al. (59)
Acetic acid, 5-[3-(4-methoxyphenyl)oxaziridin-2-yl]pentyl ester	11.215	1.954	Antibacterial	Ahmad et al. (60)
5-Hydroxymethylfurfural	11.586	33.757	Antioxidant and antimicrobial	Kaushal and Sharma (61)
Z-8-Methyl-9-tetradecenoic acid	12.583	1.105	Antibacterial	Kadhim et al. (62)
2-Propanone, 1-(4-methoxyphenyl)-	12.955	2.855	Antibacterial	Kawase et al. (63)
Geranyl isovalerate	14.380	1.653	Anticancer	Rasool et al. (64)
1,25-Dihydroxyvitamin D3	14.704	1.225	Antibacterial	Wang et al. (65)
1-(2-Hydroxy-2-methylpropyl)	16.293	2.671	Antibacterial	Sagar and Vidyasagar (66)

Among the detected constituents, 5-hydroxymethylfurfural (5-HMF) was the most abundant compound, accounting for 33.757% of the total identified metabolites with a retention time of 11.586 min. As a naturally occurring furan derivative produced during carbohydrate degradation, 5-HMF has been extensively reported to exhibit antioxidant, antimicrobial, anti-inflammatory, and cytoprotective activities. Its predominance in the extract indicates that it is likely a major contributor to the biological activities of garlic, particularly its antioxidant and antimicrobial effects.

Other major compounds identified included 3-hydroxy-2,3-dihydromaltol (7.896%), furfural (6.980%), anethole (5.000%), and 1H-azirine, octahydro-1-nitroso- (4.409%). Furfural and related furan derivatives are recognized for their antibacterial, antifungal, and antioxidant activities, whereas anethole is a well-known phenylpropanoid with antibacterial, antioxidant, anti-inflammatory, and analgesic properties. The relatively high abundance of these constituents suggests that they may act synergistically to enhance the antimicrobial activity of the garlic extract.

Compounds detected in moderate amounts included methyl furfural (3.481%), 2-propanone, 1-(4-methoxyphenyl)- (2.855%), methymaleic anhydride (2.273%), dimethyl sulfoxide (2.000%), acetic acid derivatives (1.954%), 4-hydroxymethyl-2-pyranone (1.954%), glycyl-D-threonine (1.759%), geranyl isovalerate (1.653%), nonanoic acid (1.458%), 1,25-dihydroxyvitamin D3 (1.225%), nonaldehyde (1.218%), Z-8-methyl-9-tetradecenyl acetate (1.105%), tetrahydropyran (1.042%), maleimide (1.000%), and hexadecanoic acid (1.000%). These metabolites have been associated with a broad spectrum of pharmacological activities, including antimicrobial, antioxidant, anti-inflammatory, anticancer, and immunomodulatory effects. Their combined presence may contribute to the overall biological efficacy of the extract through additive or synergistic interactions.

Minor constituents identified included 9,10-epoxyoctadecanoic acid (0.769%), β-hydroxyvaleric acid (0.323%), and 1-glutamic acid monobenzyl ester (0.313%).

#### 3.1.3 Bioactive compounds in pomegranate peel extract

Table 5 shows the GC–MS analysis of the bioactive constituents detected in the pomegranate peel extract, presenting their retention times (RT), relative peak area percentages, chemical identities, and reported biological activities. The chromatographic profile demonstrated a complex mixture of phytochemicals, including aldehydes, ketones, furans, fatty acids, heterocyclic compounds, aromatic hydrocarbons, and organic acid derivatives. According to previous studies, many of these metabolites exhibit antibacterial, antioxidant, antifungal, and antimicrobial activities, highlighting the therapeutic potential of pomegranate peel.

**Table 5:** Pharmacologically active constituents of a pomegranete peel ethanolic extract determined by GC-MS chromatography.

Composition	Retention time	% of total	Pharmacological activity	Reference
(Trimethylsilyl)ethanol	2.809	0.697	Antibacterial	Xue (67)
o-Acetyl-L-serine	3.455	0.090	Antibacterial	Parmar et al. (68)
2-Cyclohexylpiperidine	3.639	0.259	Antimicrobial	
Furfural	3.963	6.995	Antibacterial	Chai et al. (47)
6-Oxa-bicyclo[3.1.0]hexan-3-one	5.668	0.514	Antibacterial and Antifungal	Mohammed et al. (69)
2,4-Dihydroxy-2,5-dimethyl-3(2H)-furan-3-one	5.878	0.457	Antimicrobial	Enin et al. (70)
Furfural, 5-methyl	6.302	0.929	Antioxidant and antimicrobial	Rosyidah et al. (71)
Itaconic acid anhydride	6.656	1.650	Antioxidant and antimicrobial	Nayak et al. (72)
9-Oxa-bicyclo[3.3.1]nonane-1,4-diol	7.094	0.237	Antioxidant and antimicrobial	Aina and Fagbemi (73)
3-Trifluoroacetoxypentadecane	7.614	0.300	Antioxidant and antimicrobial	Uddin et al. (74)
Dodecanoic acid, 3-hydroxy	7.756	1	Antibacterial and Antifungal	Belkacemi et al. (75)
Furyl hydroxymethyl ketone	8.392	4.463	Antioxidant	Lin and Huang (76)
2,5-Furandicarboxaldehyde	8.993	1.178	Antioxidant and antimicrobial	Elsadek and Al-Numair (77)
Pyranone	9.296	5.602	Antioxidant and antimicrobial	Elaasser et al. (78)
1.4-Ethylheptane	9.596	1.570	Antimicrobial	Byju et al. (79)
2-t-Butyl-5-propyl-[1,3]dioxolan-4-one	9.778	0.419	Antioxidant and antimicrobial	Mishra et al. (80)
Melezitose	9.939	0.171	Antibacterial	Dżugan et al. (53)
1-Heptatriacotanol	10.363	0.141	Antioxidant and antimicrobial	Jasna and Khaleel (81)
2-Myristynoyl pantetheine	10.482	0.082	Antimicrobial	Sophiya et al. (82)
5-Hydroxymethylfurfural	12.209	69.628	Antioxidant and antimicrobial	Kaushal and Sharma (61)

Among the identified compounds, furfural was the most abundant constituent, representing 6.995% of the total peak area at a retention time of 3.962 min. Furfural, a furan-derived aldehyde generated during the degradation of plant polysaccharides, has been extensively documented for its antibacterial, antioxidant, antifungal, and antimicrobial properties. Its predominance in the extract suggests that it may play a significant role in the observed biological activities.

Other major constituents included 2-propanone (an acetone derivative) (5.602%), benzyl alcohol ketone (4.663%), and 1,5-pentanedicarboxaldehyde (3.175%). Aldehydes and ketones are recognized for their antimicrobial effects through disruption of microbial cell membranes and interference with essential cellular functions. Furthermore, several compounds within these chemical groups possess antioxidant properties by scavenging reactive oxygen species and reducing oxidative stress.

Compounds present at moderate levels included tetradecanoic acid anhydride (1.650%), 1,6-anhydroglucose (1.570%), 3-hydroxydodecanoic acid (1.000%), and several benzyl alcohol derivatives. Fatty acid derivatives, particularly hydroxy fatty acids, have been reported to exhibit antibacterial and antifungal activities, while carbohydrate-derived metabolites such as anhydroglucose derivatives may contribute to antioxidant capacity. The presence of these diverse phytochemicals indicates that different classes of metabolites may act synergistically, thereby enhancing the overall pharmacological properties of the extract.

In addition, several constituents were identified in relatively low abundances, including 2-cyclohexylcyclopentanone, 2,4-dihydroxy-5,5-dimethyl-2(5H)-furanone, pentanal, 5-oxo-hexyl-2,3-thiophene-1,4-dione, 2-trifluoroacetoxytridecane, 2-hydroxyethyl guanidine, maleimide, 1-heptadecanol, and 2-hydroxyethyl piperazine. Although each of these compounds accounted for less than 1% of the total extract, members of these chemical classes have previously been associated with antibacterial, antimicrobial, antioxidant, and antifungal activities. Consequently, despite their low concentrations, these metabolites may contribute to the overall biological efficacy of the pomegranate peel extract through additive or synergistic interactions.

### 3.2 Antibacterial activity of herbal extracts

**Figure 1:**
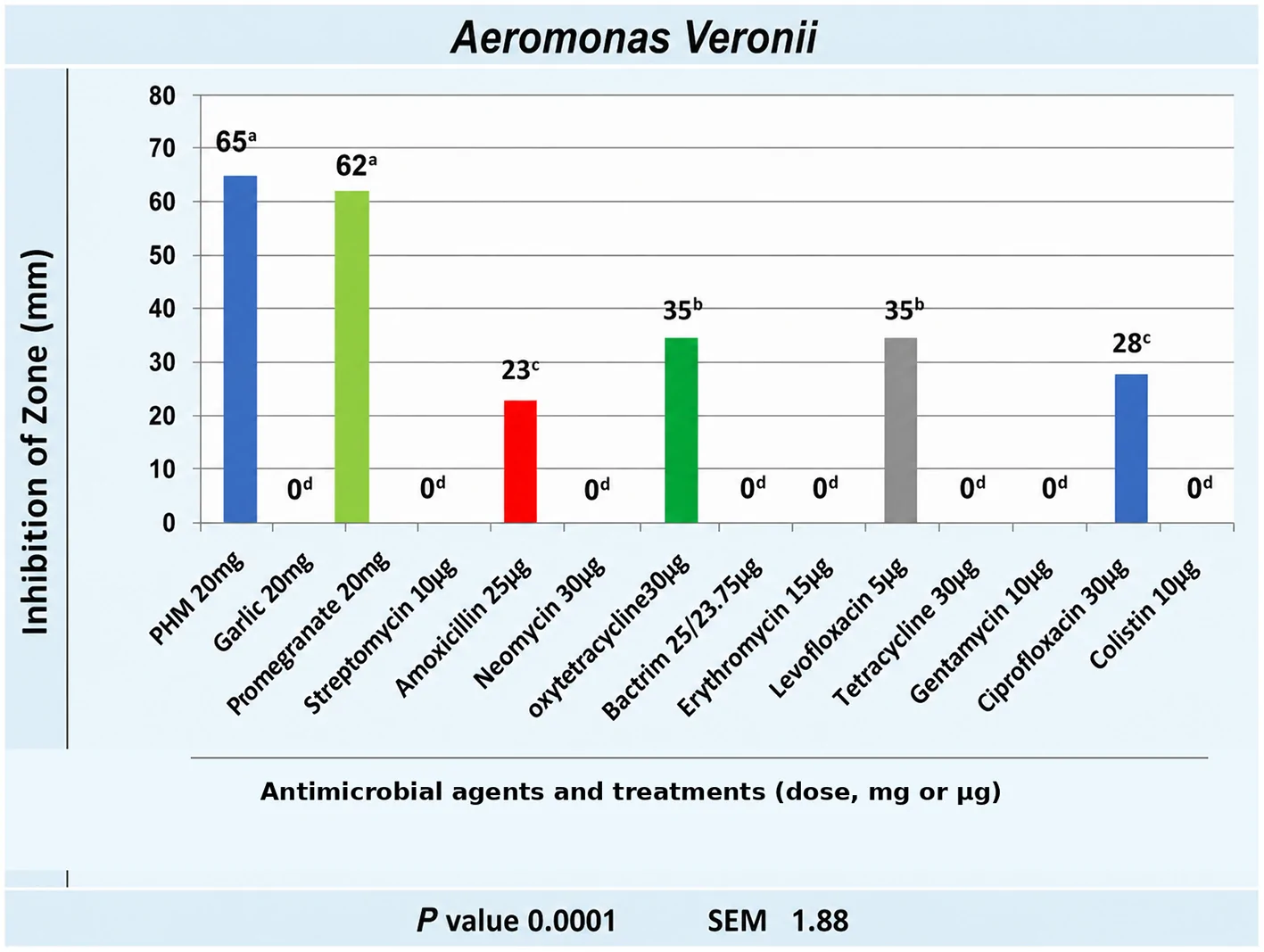
Inhibition zone of 11 different antibiotics and poly-herbal mixture (PHM), garlic, and pomegranate against *A. veronii*.

The isolated *Aeromonas veronii* strain (OM32) demonstrated multidrug resistance against several commonly used antibiotics. Among the 11 tested antibiotics, only oxytetracycline, levofloxacin, ciprofloxacin, and amoxicillin exhibited clear inhibitory activity against the isolate. The largest inhibition zones were observed for oxytetracycline and levofloxacin (35 mm each), followed by ciprofloxacin (28 mm) and amoxicillin (23 mm). In contrast, the isolate showed resistance to streptomycin, neomycin, trimethoprim-sulfamethoxazole (Bactrim), erythromycin, tetracycline, gentamicin, and colistin (Figure 1).

The ethanolic extracts of pomegranate peel and the polyherbal mixture (PHM) demonstrated notable antibacterial activity against *A. veronii*, producing inhibition zones that were significantly larger than those observed for several tested antibiotics under the experimental conditions employed (*P* < 0.05). However, because only disc diffusion assays were performed, these findings should not be interpreted as evidence of greater antimicrobial potency. Quantitative antimicrobial evaluations, including minimum inhibitory concentration (MIC), minimum bactericidal concentration (MBC), bacterial burden assessments, and dose–response analyses, are required before conclusions regarding relative efficacy or antimicrobial potency can be established.

### 3.3 Clinical signs and mortality rate

**Figure 2:**
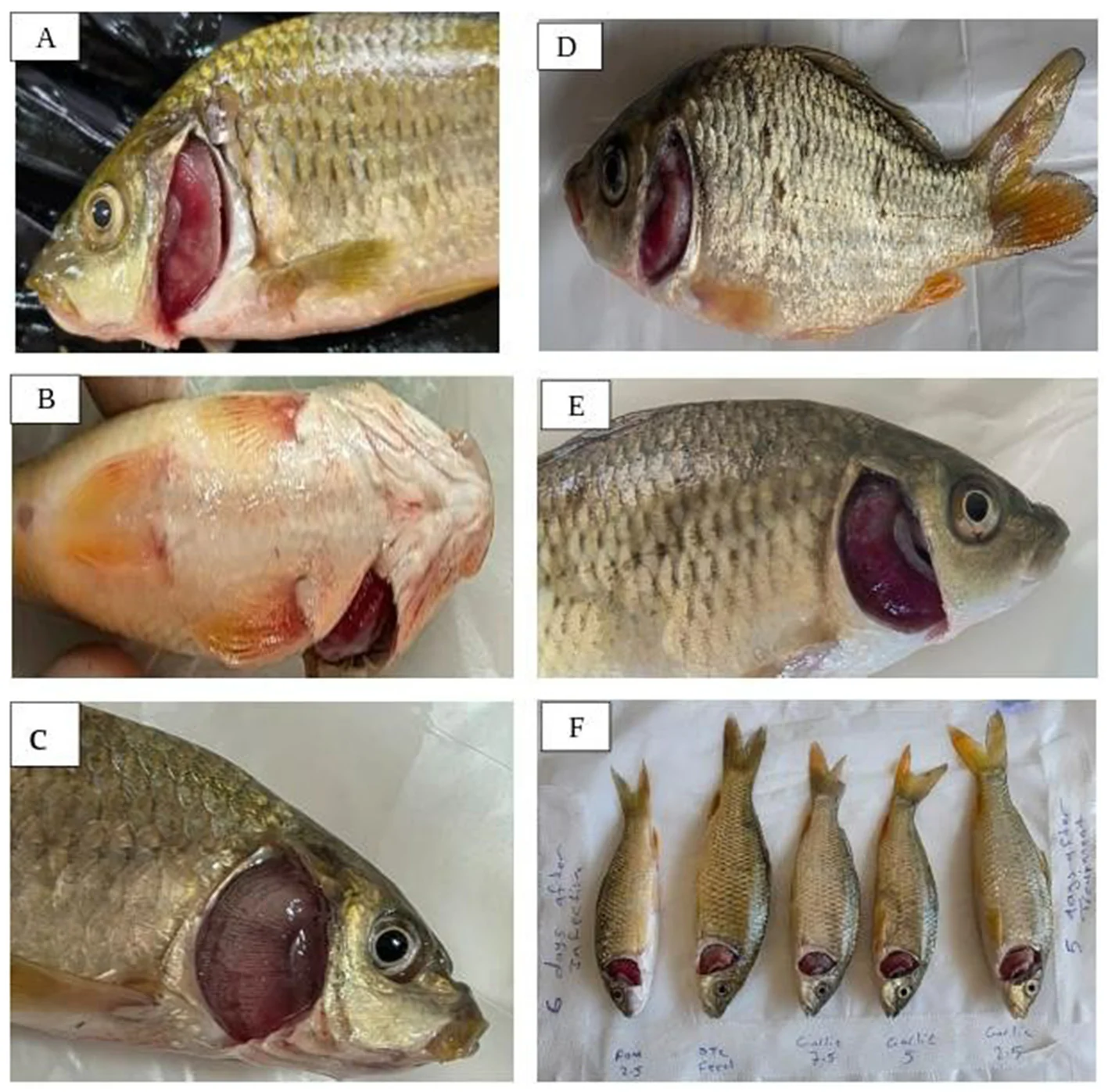
Symptoms of external organs and gill lesions in infected common carp fish varied with the days of infection and treatments in those challenged with *Aeromonas veronii*. After 1 day of infection, the gills change color, appearing pink or gray due to ischemia **(A)**. By the second day, hemorrhagic symptoms from the operculum extend to the abdominal sites of the body fish **(B)**. Black necrotic areas were seen in the base of the gill tissue of untreated infected fish after 3 days, and the gill filaments were sloughing **(C)**. Experimental infected fish after 4 and 5 days led to severe gill lesions and deteriorated gill tissue (**D** and **E**). *Aeromonas veronii* causes the death of infected common carp fish that were treated with Garlic in different doses (2.5, 5, and 7.5%), 2.5% of pomegranate, and OTC by feed for 6 days **(F)**.

Fish experimentally infected with *A. veronii* exhibited severe clinical signs shortly after exposure. The earliest signs included lethargy, reduced feeding activity, abnormal swimming behavior, excessive mucus secretion, and hemorrhages around the gill region. As the infection progressed, fish developed severe gill lesions characterized by epithelial sloughing, necrotic areas, and extensive tissue destruction. Abdominal distension and respiratory distress were also observed in heavily infected fish (Figure 2).

Mortality rates differed significantly among experimental groups (*P* < 0.05; Table 6). During the first 5 days post-infection, the untreated infected group exhibited the highest cumulative mortality rate, reaching approximately 77.8%. Mortality continued to increase throughout the experimental period, ultimately reaching 100% by day 14.

**Table 6:** Effect of garlic, pomegranate peel, PHM, or oxytetracycline on mortality rate% in common carp infected with *A. veronii*.

Treatments	1–5 days	6–10 days	1–10 days	11–14 days	1–14 days
Control	0.00 ± 4.765^c^	0.00 ± 5.928^b^	0.00 ± 6.234^f^	0.00 ± 7.374^b^	0.00 ± 7.348^c^
Infected	77.78 ± 4.765^a^	33.34 ± 5.928^ab^	88.89 ± 6.234^ab^	100 ± 7.374^a^	100 ± 7.348^a^
OCT feed	16.67 ± 4.765^bc^	30.00 ± 5.928^ab^	33.33 ± 6.234^b − f^	8.33 ± 7.374^b^	36.67 ± 7.348^b^
OCT bath	16.67 ± 4.765^bc^	0.00 ± 5.928^b^	16.67 ± 6.234^d − f^	13.13 ± 7.374^b^	16.68 ± 7.348^bc^
Garlic 2.5%	50.0 ± 4.765^ab^	50.0 ± 5.928^ab^	66.67 ± 6.234^a − e^	100 ± 7.374^a^	100 ± 7.348^a^
Garlic 5%	50.0 ± 4.765^ab^	10.0 ± 5.928^b^	55.55 ± 6.234^a − f^	100 ± 7.374^a^	100 ± 7.348^a^
Garlic 7.5%	22.22 ± 4.765^bc^	0.00 ± 5.928^b^	22.22 ± 6.234^c − f^	100 ± 7.374^a^	100 ± 7.348^a^
PomP 2.5%	27.78 ± 4.765^abc^	68.33 ± 5.928^ab^	77.78 ± 6.234^a − c^	0.00 ± 7.374^b^	77.76 ± 7.348^a^
PomP 5%	44.44 ± 4.765^ab^	91.67 ± 5.928^a^	94.44 ± 6.234^a^	0.00 ± 7.374^b^	94.43 ± 7.348^a^
PomP 7.5%	50.0 ± 4.765^ab^	38.89 ± 5.928^ab^	72.22 ± 6.234^a − d^	66.67 ± 7.374^a^	88.87 ± 7.348^a^
PHM 2.5%	11.11 ± 4.765^bc^	0.00 ± 5.928^b^	11.11 ± 6.234^ef^	0.00 ± 7.374^b^	11.1 ± 7.348^bc^
PHM 5%	11.11 ± 4.765^bc^	0.00 ± 5.928^b^	11.11 ± 6.234^ef^	0.00 ± 7.374^b^	11.1 ± 7.348^bc^
PHM 7.5%	5.56 ± 4.765^c^	0.00 ± 5.928^b^	5.56 ± 6.234^f^	0.00 ± 7.374^b^	5.57 ± 7.348^c^
	Probability				
*P* value	0.0051	0.0002	0.0001	0.0001	0.0001

OCT, oxytetracycline; PomP, pomegranate peel; PHM, poly-herbal mixture.

a − f Different letters in the same column show a significant difference at *P* < 0.05.

The selected sampling points (7 and 14 days post-infection) were designed to evaluate intermediate and late infection responses. Earlier stages of infection were not investigated in the present study.

Garlic supplementation at all tested concentrations (2.5%, 5%, and 7.5%) failed to protect fish against *A. veronii* infection, with all garlic-treated groups reaching 100% mortality by the end of the experiment. Similarly, pomegranate peel supplementation showed limited protection, particularly at lower concentrations.

In contrast, fish supplemented with PHM exhibited markedly improved survival rates. The 7.5% polyherbal mixture (PHM) group showed the lowest cumulative mortality (5.57%), corresponding to approximately 94% survival. Mortality rates in the PHM-treated groups were significantly lower than those observed in the oxytetracycline-treated groups under the conditions of this experiment (*P* < 0.05). Oxytetracycline administered in feed and bath treatments reduced cumulative mortality to 36.67 and 16.68%, respectively.

### 3.4 Differential leukocyte counts

The effects of dietary treatments on leukocyte profiles at 7 and 14 days post-infection are presented in Tables 7, 8.

**Table 7:** Effect of the garlic, pomegranate peel, PHM, or oxytetracycline on differential leukocytes% in common carp infected by *A. veronii* at 7 days post-infection.

Treatments	Neutrophile	Lymphocyte	Monocyte	Basophile	Eosinophile
Control	33.47 ± 1.661^g^	58.83 ± 1.527^a^	4.20 ± 0.364^abc^	2.13 ± 0.228	1.37 ± 0.095
Infected	55.60 ± 1.661^b − d^	36.73 ± 1.527^c − f^	4.67 ± 0.364^abc^	2.10 ± 0.228	0.90 ± 0.095
OCT feed	53.40 ± 1.661^c − e^	42.13 ± 1.527^b − d^	3.57 ± 0.364^abc^	0.67 ± 0.228	0.23 ± 0.095
OCT bath	59.90 ± 1.661^a − c^	34.13 ± 1.527^d − f^	4.67 ± 0.364^abc^	1.03 ± 0.228	0.27 ± 0.095
Garlic 2.5%	60.60 ± 1.661^a − c^	29.77 ± 1.527^f^	7.93 ± 0.364^a^	1.27 ± 0.228	0.43 ± 0.095
Garlic 5%	66.13 ± 1.661^a^	32.20 ± 1.527^d − f^	0.83 ± 0.364^c^	0.63 ± 0.228	0.20 ± 0.095
Garlic 7.5%	63.67 ± 1.661^ab^	30.87 ± 1.527^ef^	3.70 ± 0.364^abc^	1.33 ± 0.228	0.43 ± 0.095
PomP 2.5%	46.40 ± 1.661^d − f^	49.17 ± 1.527^ab^	2.37 ± 0.364^bc^	1.50 ± 0.228	0.57 ± 0.095
PomP 5%	53.20 ± 1.661^c − e^	40.97 ± 1.527^b − e^	3.20 ± 0.364^abc^	2.03 ± 0.228	0.60 ± 0.095
PomP 7.5%	63.73 ± 1.661^ab^	30.43 ± 1.527^f^	4.63 ± 0.364^abc^	0.60 ± 0.228	0.60 ± 0.095
PHM 2.5%	43.90 ± 1.661^ef^	48.53 ± 1.527^ab^	5.27 ± 0.364^abc^	1.77 ± 0.228	0.53 ± 0.095
PHM 5%	44.60 ± 1.661^ef^	45.07 ± 1.527^bc^	6.27 ± 0.364^ab^	3.47 ± 0.228	0.60 ± 0.095
PHM 7.5%	39.97 ± 1.661^fg^	51.13 ± 1.527^ab^	6.07 ± 0.364^ab^	1.90 ± 0.228	0.93 ± 0.095
	Probability				
*P* value	0.0001	0.0001	0.0061	0.5415	0.6044

OCT, oxytetracycline; PomP, pomegranate peel; PHM, poly-herbal mixture.

a − g Different letters in the same column show a significant difference at *P* < 0.05.

**Table 8:** Effect of the garlic, pomegranate peel, PHM, or oxytetracycline on differential leukocytes% in common carp infected by *A. veronii* at 14 days post-infection.

Treatments	Neutrophile	Lymphocyte	Monocyte	Basophile	Eosinophile
Control	33.30 ± 0.383^c^	55.40 ± 0.592^bc^	7.10 ± 0.300^a^	2.33 ± 0.173	1.83 ± 0.192^ab^
OCT feed	35.83 ± 0.383^abc^	54.87 ± 0.592^bc^	4.57 ± 0.300^ab^	2.00 ± 0.173	2.73 ± 0.192^a^
OCT bath	34.57 ± 0.383^bc^	59.17 ± 0.592^abc^	3.10 ± 0.300^b^	1.53 ± 0.173	1.63 ± 0.192^ab^
PomP 2.5%	37.03 ± 0.383^ab^	54.50 ± 0.592^c^	5.17 ± 0.300^ab^	1.17 ± 0.173	1.67 ± 0.192^ab^
PomP 5%	34.53 ± 0.383^bc^	58.87 ± 0.592^abc^	4.73 ± 0.300^ab^	2.13 ± 0.173	0.70 ± 0.192^ab^
PomP 7.5%	36.23 ± 0.383^abc^	55.50 ± 0.592^bc^	5.83 ± 0.300^ab^	1.33 ± 0.173	1.10 ± 0.192^ab^
PHM 2.5%	38.40 ± 0.383^a^	56.10 ± 0.592^bc^	4.13 ± 0.300^ab^	1.33 ± 0.173	0.23 ± 0.192^b^
PHM 5%	33.80 ± 0.383^bc^	60.20 ± 0.592^ab^	3.77 ± 0.300^ab^	1.37 ± 0.173	0.87 ± 0.192^ab^
PHM 7.5%	33.20 ± 0.383^c^	62.10 ± 0.592^a^	3.57 ± 0.300^b^	0.39 ± 0.173	0.20 ± 0.192^b^
	Probability				
*P* value	0.0006	0.0009	0.0199	0.5675	0.0140

OCT, Oxytetracycline; PomP, Pomegranate peel; PHM, Poly-herbal mixture.

a − c Different letters in the same column show a significant difference at *P* < 0.05.

At 7 days post-infection, *A. veronii* infection significantly increased neutrophil percentages and reduced lymphocyte levels compared with the control group (*P* < 0.05). The untreated infected group exhibited pronounced neutrophilia and lymphocytopenia, indicating an acute inflammatory response. Dietary supplementation with polyherbal mixture (PHM) significantly reduced neutrophil percentages while restoring lymphocyte levels toward normal values. Fish receiving 7.5% PHM showed leukocyte profiles most similar to the control group.

Garlic-treated groups exhibited the highest neutrophil counts and the lowest lymphocyte levels among all treatments, reflecting severe systemic infection and poor physiological recovery. Monocyte counts were significantly altered in some treatment groups, whereas eosinophil and basophil percentages were not significantly affected.

At 14 days post-infection, no hematological data were available for untreated infected fish or garlic-treated groups because of complete mortality. Fish supplemented with 7.5% PHM maintained significantly higher lymphocyte percentages and lower neutrophil counts compared with other infected groups (*P* < 0.05), indicating an association with improved leukocyte profile recovery during infection.

### 3.5 Hematological parameters

The hematological responses of common carp following *A. veronii* challenge are summarized in Table 9. Infection caused marked reductions in red blood cell count (RBC), hemoglobin concentration (Hb), and haematocrit (HCT), indicating severe anemia and impaired oxygen-carrying capacity in infected fish.

**Table 9:** Effect of the garlic, pomegranate peel, PHM, or oxytetracycline on hematological parameters in common carp infected by *A. veronii* at 14 days post-infection.

Treatments	HCT %	Hb mg/dL	RBCs cell × 10^6^/mL^3^	MCV fL	MCH Pg/cell	MCHC mg/dL
Control	31.0 ± 1.287^a^	9.53 ± 0.512^a^	1.633 ± 0.122^a^	185 ± 9.608^a^	59 ± 3.843^a^	26.8 ± 0.769^a^
OCT feed	17.0 ± 1.287^cde^	3.93 ± 0.512^cd^	0.233 ± 0.122^cd^	80 ± 9.608^cd^	17 ± 3.843^cd^	18.4 ± 0.769^cd^
OCT bath	20.3 ± 1.287^c^	5.27 ± 0.512^c^	0.567 ± 0.122^c^	105 ± 9.608^c^	27 ± 3.843^c^	20.4 ± 0.769^c^
PomP 2.5%	12.7 ± 1.287^e^	2.33 ± 0.512^d^	0.027 ± 0.122^d^	50 ± 9.608^d^	5 ± 3.843^d^	16.0 ± 0.769^d^
PomP 5%	15.3 ± 1.287^de^	3.13 ± 0.512^d^	0.067 ± 0.122^d^	65 ± 9.608^d^	11 ± 3.843^d^	17.2 ± 0.769^d^
PomP 7.5%	19.7 ± 1.287^cd^	5.00 ± 0.512^c^	0.500 ± 0.122^c^	100 ± 9.608^c^	25 ± 3.843^c^	20.0 ± 0.769^c^
PHM 2.5%	26.3 ± 1.287^b^	7.67 ± 0.512^b^	1.167 ± 0.122^b^	150 ± 9.608^b^	45 ± 3.843^b^	24.0 ± 0.769^b^
PHM 5%	28.3 ± 1.287^ab^	8.47 ± 0.512^ab^	1.367 ± 0.122^ab^	165 ± 9.608^ab^	51 ± 3.843^ab^	25.2 ± 0.769^ab^
PHM 7.5%	30.3 ± 1.287^ab^	9.27 ± 0.512^ab^	1.567 ± 0.122^a^	180 ± 9.608^ab^	57 ± 3.843^ab^	26.4 ± 0.769^ab^
	Probability					
*P* value	0.0001	0.0001	0.0001	0.0001	0.0001	0.0001

HCT, haematocrit; Hb, hemoglobin; RBCs, red blood cells; MCV, mean corpuscular volume; MCH, mean corpuscular hemoglobin; MCHC, mean corpuscular hemoglobin concentration; OCT, oxytetracycline; PomP, pomegranate peel; PHM, poly-herbal mixture.

a − e Different letters in the same column show a significant difference at *P* < 0.05.

The untreated infected group showed the greatest deterioration in hematological indices, whereas PHM supplementation significantly ameliorated these changes (*P* < 0.05). Fish receiving diets supplemented with 5 and 7.5% PHM maintained RBC, Hb, and HCT values closer to those of the control group. Similarly, mean corpuscular volume (MCV), mean corpuscular hemoglobin (MCH), and mean corpuscular hemoglobin concentration (MCHC) remained relatively stable in PHM-treated fish compared with other infected groups.

These findings indicate improved maintenance of hematological parameters during infection. However, direct indicators of erythropoietic activity were not evaluated. Preservation of normal RBC indices in PHM-treated fish may have contributed to improved oxygen transport and overall physiological stability during infection.

In contrast, pomegranate peel and oxytetracycline treatments provided only partial improvement in hematological parameters, while garlic-treated groups exhibited severe hematological deterioration before mortality.

### 3.6 Liver enzyme activities

The effects of dietary treatments on liver enzyme activities are shown in Table 10. Infection with *A. veronii* significantly elevated plasma alanine aminotransferase (ALT) and alkaline phosphatase (ALP) levels at both 7 and 14 days post-infection compared with the control group (*P* < 0.05), indicating hepatic stress and tissue damage.

**Table 10:** Effect of the garlic, pomegranate peel, PHM, or oxytetracycline on Liver enzymes in common carp infected by *A. veronii* at 7- and 14-days post-infection.

Treatments	7 days		14 days	
	ALT (IU/L)	ALP (IU/L)	ALT (IU/L)	ALP (IU/L)
Control	71.3 ± 11.776^c^	69.3 ± 6.078^e − g^	80.7 ± 7.327^cde^	33.0 ± 9.672^h^
Infected	290.3 ± 11.776^a^	155.0 ± 6.078^ab^		
OCT feed	311.0 ± 11.776^a^	164.0 ± 6.078^a^	79.3 ± 7.327^cde^	78.3 ± 9.672^fg^
OCT bath	177.0 ± 11.776^c^	125.3 ± 6.078^a − c^	85.0 ± 7.327^cd^	135.0 ± 9.672^c^
Garlic 2.5%	303.7 ± 11.776^a^	98.3 ± 6.078^c − f^		
Garlic 5%	197.7 ± 11.776^bc^	43.0 ± 6.078^g^		
Garlic 7.5%	312.7 ± 11.776^a^	117.7 ± 6.078^b − d^		
PomP 2.5%	299.0 ± 11.776^a^	110.0 ± 6.078^c − e^	138.7 ± 7.327^a^	82.7 ± 9.672^f^
PomP 5%	265.7 ± 11.776^a^	80.3 ± 6.078^e − g^	69.7 ± 7.327^de^	71.0 ± 9.672^g^
PomP 7.5%	258.0 ± 11.776^ab^	67.7 ± 6.078^e − g^	113.0 ± 7.327^b^	150.0 ± 9.672^b^
PHM 2.5%	181.3 ± 11.776^c^	81.7 ± 6.078^c − g^	93.0 ± 7.327^bc^	111.0 ± 9.672^d^
PHM 5%	196.3 ± 11.776^c^	124.3 ± 6.078^a − d^	66.3 ± 7.327^de^	179.3 ± 9.672^a^
PHM 7.5%	173.3 ± 11.776^c^	60.0 ± 6.078^fg^	61.0 ± 7.327^e^	96.0 ± 9.672^e^
	Probability			
*P* value	0.0001	0.0001	0.0001	0.0001

ALT, alanine transaminase; ALP, alkaline phosphatase; OCT, oxytetracycline; PomP, pomegranate peel; PHM, poly-herbal mixture.

a − g Different letters in the same column show a significant difference at *P* < 0.05.

PHM supplementation significantly reduced ALT and ALP levels compared with untreated infected fish. The greatest improvement was observed in fish receiving 5 and 7.5% PHM, where enzyme activities approached values comparable to those of the control group. Oxytetracycline and pomegranate peel treatments also reduced liver enzyme elevations but were generally less effective than PHM supplementation.

Although reductions in ALT and ALP activities indicate improved physiological status, oxidative stress biomarkers such as superoxide dismutase (SOD), catalase (CAT), and malondialdehyde (MDA) were not evaluated. Therefore, antioxidant-related interpretations cannot be confirmed from the present data.

Garlic-treated groups demonstrated persistently elevated liver enzyme activities, reflecting severe systemic infection and liver dysfunction.

### 3.7 Gill histopathology

Severe histopathological alterations were observed in the gills of *A. veronii*-infected fish. Lesions included epithelial hyperplasia, chloride cell proliferation, mucus cell hyperplasia, lamellar fusion, epithelial lifting, hemorrhage, inflammatory cell infiltration, degeneration, and necrosis of primary and secondary gill lamellae.

Representative photomicrographs illustrating the major histopathological alterations observed among treatment groups are provided in Supplementary Figures S1–S21.

At 7 days post-infection, the untreated infected group exhibited extensive gill tissue damage characterized by severe necrosis, epithelial degeneration, and inflammatory infiltration. Garlic-treated groups showed similar pathological changes with minimal evidence of tissue recovery.

Fish treated with pomegranate peel and polyherbal mixture (PHM) demonstrated significantly improved gill architecture compared with untreated infected fish (*P* < 0.05). Among all treatments, 7.5% PHM supplementation was associated with the greatest histological improvement, with marked reductions in epithelial degeneration, necrosis, hyperplasia, and inflammatory lesions. Gill morphology in the PHM-treated groups closely resembled that of the non-infected control group (Tables 11, 12).

**Table 11:** Effect of the garlic, pomegranate peel, PHM, or oxytetracycline on gills histopathology in common carp infected by *A. veronii* at 7 days post-infection.

Treatments	Deg and Nec of PF	Deg and Nec of SF	Hyperplasia of EPCs	Hyperplasia of ChCs	Hyperplasia of MCs
Control	0.00 ± 0.192^f^	0.00 ± 0.199^e^	0.00 ± 0.187^e^	0.00 ± 0.225^g^	0.00 ± 0.258^c^
Infected	5.00 ± 0.192^a^	4.75 ± 0.199^a^	5.00 ± 0.187^a^	5.00 ± 0.225^a^	5.00 ± 0.258^a^
OCT feed	2.75 ± 0.192^de^	2.25 ± 0.199^cd^	2.75 ± 0.187^c^	3.00 ± 0.225^c^	0.50 ± 0.258^c^
OCT bath	3.75 ± 0.192^bc^	4.00 ± 0.199^ab^	2.50 ± 0.187^c^	2.50 ± 0.225^cd^	3.00 ± 0.258^b^
Garlic 2.5%	4.00 ± 0.192^b^	2.25 ± 0.199^cd^	3.00 ± 0.187^bc^	3.00 ± 0.225^c^	1.00 ± 0.258^c^
Garlic 5%	5.00 ± 0.192^a^	5.00 ± 0.199^a^	4.25 ± 0.187^a^	5.00 ± 0.225^a^	5.00 ± 0.258^a^
Garlic 7.5%	4.25 ± 0.192^ab^	4.75 ± 0.199^a^	4.00 ± 0.187^ab^	4.00 ± 0.225^b^	4.00 ± 0.258^ab^
PomP 2.5%	3.00 ± 0.192^cd^	3.00 ± 0.199^bc^	2.50 ± 0.187^c^	3.00 ± 0.225^c^	3.00 ± 0.258^b^
PomP 5%	2.75 ± 0.192^de^	3.00 ± 0.199^bc^	2.00 ± 0.187^cd^	1.50 ± 0.225^e^	0.50 ± 0.258^c^
PomP 7.5%	2.50 ± 0.192^de^	2.75 ± 0.199^cd^	2.75 ± 0.187^c^	0.25 ± 0.225^fg^	0.25 ± 0.258^c^
PHM 2.5%	2.00 ± 0.192^e^	2.00 ± 0.199^cd^	2.25 ± 0.187^cd^	1.75 ± 0.225^de^	0.50 ± 0.258^c^
PHM 5%	2.00 ± 0.192^e^	1.75 ± 0.199^d^	1.25 ± 0.187^d^	1.25 ± 0.225^e^	0.25 ± 0.258^c^
PHM 7.5%	2.00 ± 0.192^e^	2.00 ± 0.199^cd^	1.25 ± 0.187^d^	1.00 ± 0.225^ef^	1.00 ± 0.258^c^
	Probability-				
*P* value	0.0001	0.0001	0.0001	0.0001	0.0001

Deg, degeneration; Nec, necrosis; PF, primary filament; SF, secondary filament; EPCs, epithelial cells; ChCs, chloride cells; MCs, mucus cells; OCT, oxytetracycline; PomP, pomegranate peel; PHM, poly-herbal mixture.

a − f Different letters in the same column show a significant difference at *P* < 0.05.

**Table 12:** Effect of the garlic, pomegranate peel, PHM, or oxytetracycline on gills histopathology in common carp infected by *A. veronii* at 7 days post-infection.

Treatments	Lifting of SF	Clubbing of SF	Congestion or hemorrhage of the gill parenchyma	Inflammation	Oedema	Sloughing of SF
Control	0.00 ± 0.126^d^	0.00 ± 0.133^c^	0.00 ± 0.197^g^	0.00 ± 0.179^e^	0.00 ± 0.201^d^	0.00 ± 0.232^d^
Infected	0.00 ± 0.126^d^	0.00 ± 0.133^c^	4.75 ± 0.197^a^	4.50 ± 0.179^a^	0.00 ± 0.201^d^	4.75 ± 0.232^a^
OCT feed	2.25 ± 0.126^a^	2.00 ± 0.133^a^	2.00 ± 0.197^cde^	0.75 ± 0.179^de^	3.25 ± 0.201^b^	0.00 ± 0.232^d^
OCT bath	2.00 ± 0.126^a^	0.50 ± 0.133^bc^	2.00 ± 0.197^cde^	1.00 ± 0.179^cd^	0.25 ± 0.201^d^	0.00 ± 0.232^d^
Garlic 2.5%	2.00 ± 0.126^a^	2.00 ± 0.133^a^	2.50 ± 0.197^cd^	1.75 ± 0.179^c^	3.00 ± 0.201^b^	2.00 ± 0.232^c^
Garlic 5%	0.00 ± 0.126^d^	0.00 ± 0.133^c^	3.00 ± 0.197^bc^	3.00 ± 0.179^b^	0.00 ± 0.201^d^	4.75 ± 0.232^a^
Garlic 7.5%	0.00 ± 0.126^d^	0.00 ± 0.133^c^	4.00 ± 0.197^ab^	3.00 ± 0.179^b^	4.00 ± 0.201^a^	4.50 ± 0.232^a^
PomP 2.5%	0.00 ± 0.126^d^	2.50 ± 0.133^a^	2.00 ± 0.197^cde^	1.00 ± 0.179^cd^	0.00 ± 0.201^d^	1.75 ± 0.232^c^
PomP 5%	1.75 ± 0.126^ab^	2.00 ± 0.133^a^	1.00 ± 0.197^efg^	1.00 ± 0.179^cd^	0.00 ± 0.201^d^	3.00 ± 0.232^b^
PomP 7.5%	1.00 ± 0.126^bc^	1.00 ± 0.133^b^	1.75 ± 0.197^def^	1.00 ± 0.179^cd^	1.00 ± 0.201^c^	2.00 ± 0.232^c^
PHM 2.5%	0.50 ± 0.126^cd^	2.00 ± 0.133^a^	0.50 ± 0.197^g^	1.00 ± 0.179^cd^	0.00 ± 0.201^d^	2.00 ± 0.232^c^
PHM 5%	0.50 ± 0.126^cd^	0.75 ± 0.133^bc^	0.75 ± 0.197^fg^	0.50 ± 0.179^de^	0.00 ± 0.201^d^	1.25 ± 0.232^c^
PHM 7.5%	0.50 ± 0.126^cd^	0.25 ± 0.133^bc^	0.50 ± 0.197^g^	0.25 ± 0.179^de^	0.00 ± 0.201^d^	2.00 ± 0.232^c^
	Probability					
*P* value	0.0001	0.0001	0.0001	0.0001	0.0001	0.0001

SF, secondary filament; OCT, oxytetracycline; PomP, pomegranate peel; PHM, poly-herbal mixture.

a − g Different letters in the same column show a significant difference at *P* < 0.05.

At 14 days post-infection, surviving fish in the PHM and pomegranate peel groups exhibited substantial healing and regeneration of gill tissues. Histopathological scores were significantly lower than those observed in oxytetracycline-treated fish, suggesting enhanced protective and restorative effects under the conditions of this study and favorable histopathological outcomes associated with PHM supplementation against *A. veronii*-induced gill damage (Tables 13, 14).

**Table 13:** Effect of the garlic, pomegranate peel, PHM, or oxytetracycline on gills histopathology in common carp infected by *A. veronii* at 14 days post-infection.

Treatments	Deg and Nec of PF	Deg and Nec of SF	Hyperplasia of EPCs	Hyperplasia of ChCs	Hyperplasia of MCs
Control	0.00 ± 0.196^d^	0.00 ± 0.209^d^	0.00 ± 0.187^e^	0.00 ± 0.106^c^	0.00 ± 0.113^b^
OTC feed	4.00 ± 0.196^a^	4.00 ± 0.209^a^	4.00 ± 0.187^a^	1.50 ± 0.106^ab^	0.50 ± 0.113^b^
OTC bath	3.75 ± 0.196^a^	4.00 ± 0.209^a^	2.00 ± 0.187^bc^	1.00 ± 0.106^b^	0.50 ± 0.113^b^
PomP 2.5%	2.75 ± 0.196^b^	2.75 ± 0.209^b^	1.75 ± 0.187^bc^	1.00 ± 0.106^b^	0.25 ± 0.113^b^
PomP 5%	2.00 ± 0.196^c^	1.75 ± 0.209^c^	2.25 ± 0.187^b^	2.00 ± 0.106^a^	0.75 ± 0.113^b^
PomP 7.5%	2.00 ± 0.196^c^	1.75 ± 0.209^c^	2.25 ± 0.187^b^	2.00 ± 0.106^a^	2.00 ± 0.113^a^
PHM 2.5%	1.50 ± 0.196^c^	1.50 ± 0.209^c^	1.75 ± 0.187^bc^	1.50 ± 0.106^ab^	0.75 ± 0.113^b^
PHM 5%	2.00 ± 0.196^c^	1.75 ± 0.209^c^	1.25 ± 0.187^cd^	1.00 ± 0.106^b^	0.50 ± 0.113^b^
PHM 7.5%	2.00 ± 0.196^c^	2.00 ± 0.209^bc^	0.75 ± 0.187^de^	1.00 ± 0.106^b^	0.75 ± 0.113^b^
	Probability				
*P* value	0.0001	0.0001	0.0001	0.0001	0.0003

Deg, degeneration; Nec, necrosis; PF, primary filament; SF, secondary filament; EPCs, epithelial cells; ChCs, chloride cells; MCs, mucus cells; OCT, oxytetracycline; PomP, pomegranate peel; PHM, poly-herbal mixture.

a − e Different letters in the same column show a significant difference at *P* < 0.05.

**Table 14:** Effect of the garlic, pomegranate peel, PHM, or oxytetracycline on gills histopathology in common carp infected by *A. veronii* at 14 days post-infection.

Treatments	Lifting of SF	Clubbing of SF	Congestion or hemorrhage of the gill parenchyma	Inflammation	Oedema	Sloughing of SF
Control	0.00 ± 0.081	0.00 ± 0.115^c^	0.00 ± 0.222^d^	0.00 ± 0.151^d^	0.00 ± 0.053^b^	0.00 ± 0.104^b^
OTC feed	0.00 ± 0.081	0.00 ± 0.115^c^	4.25 ± 0.222^a^	3.00 ± 0.151^a^	0.25 ± 0.053^ab^	0.00 ± 0.104^b^
OTC bath	0.25 ± 0.081	2.00 ± 0.115^a^	1.00 ± 0.222^c^	1.00 ± 0.151^bc^	0.00 ± 0.053^b^	0.00 ± 0.104^b^
PomP 2.5%	0.75 ± 0.081	0.75 ± 0.115^b^	2.00 ± 0.222^b^	1.00 ± 0.151^bc^	0.00 ± 0.053^b^	1.00 ± 0.104^a^
PomP 5%	0.50 ± 0.081	0.00 ± 0.115^c^	2.00 ± 0.222^b^	1.25 ± 0.151^b^	0.75 ± 0.053^a^	1.50 ± 0.104^a^
PomP 7.5%	0.75 ± 0.081	0.75 ± 0.115^b^	0.50 ± 0.222^cd^	0.75 ± 0.151^bcd^	0.00 ± 0.053^b^	1.00 ± 0.104^a^
PHM 2.5%	0.50 ± 0.081	0.00 ± 0.115^c^	0.50 ± 0.222^cd^	0.50 ± 0.151^bcd^	0.00 ± 0.053^b^	1.00 ± 0.104^a^
PHM 5%	0.25 ± 0.081	0.00 ± 0.115^c^	0.50 ± 0.222^cd^	0.50 ± 0.151^bcd^	0.00 ± 0.053^b^	1.00 ± 0.104^a^
PHM 7.5%	0.25 ± 0.081	0.00 ± 0.115^c^	0.25 ± 0.222^cd^	0.25 ± 0.151^cd^	0.00 ± 0.053^b^	0.75 ± 0.104^ab^
	Probability					
*P* value	0.2036	0.0001	0.0001	0.0001	0.0012	0.0001

SF, secondary filament; OCT, oxytetracycline; PomP, pomegranate peel;PHM, poly-herbal mixture.

a − d Different letters in the same column show a significant difference at *P* < 0.05.

## 4 Discussion

### 4.1 Effects of PHM in *Aeromonas veronii*-infected *Cyprinus carpio*

*Aeromonas veronii* is an important Gram-negative opportunistic pathogen responsible for severe bacterial diseases in freshwater fish, causing substantial economic losses to the aquaculture industry worldwide (25, 26). In the present study, experimental infection with *A. veronii* resulted in severe gill lesions, hematological disturbances, liver dysfunction, and almost complete mortality in common carp, confirming the high virulence of the isolate. In contrast, dietary supplementation with the polyherbal mixture (PHM), particularly at the 7.5% inclusion level, markedly improved survival, hematological status, liver enzyme profiles, and gill tissue integrity. Collectively, these findings indicate that PHM may represent a promising complementary strategy for improving disease resistance and reducing dependence on conventional antibiotics in aquaculture.

The isolated *A. veronii* strain exhibited multidrug resistance to several commonly used antibiotics, including streptomycin, neomycin, gentamicin, erythromycin, tetracycline, and colistin, consistent with previous reports describing the increasing prevalence of antimicrobial-resistant *Aeromonas spp*. in aquaculture (27, 28). This trend is of increasing concern because antimicrobial resistance threatens both sustainable fish production and public health (29–31). Although oxytetracycline remained effective against the present isolate, tetracycline showed considerably lower activity, which may reflect differences in bacterial efflux systems and ribosomal binding affinity associated with tetracycline-resistance genes. Interestingly, PHM and pomegranate peel extract produced larger inhibition zones than several tested antibiotics under the experimental conditions employed, suggesting substantial antibacterial activity against *A. veronii*. Nevertheless, disc diffusion assays provide only preliminary evidence of antimicrobial activity and cannot accurately determine antimicrobial potency or distinguish bacteriostatic from bactericidal effects. Therefore, future investigations should include MIC, MBC, bacterial burden quantification, and time-kill kinetic analyses to better characterize the antimicrobial properties of PHM. Furthermore, because biofilm formation is an important virulence mechanism in *A. veronii*, evaluating the effects of PHM on biofilm formation, biofilm eradication, and biofilm-associated virulence factors would provide valuable mechanistic insight into its antibacterial activity.

The protective effects of PHM are likely attributable to the complementary biological activities of its constituent medicinal plants, including black cumin, pomegranate peel, rosemary, fenugreek, sesame, peppermint, and thyme, all of which contain compounds with reported antimicrobial, anti-inflammatory, antioxidant, and immunomodulatory properties (1–4, 6, 7). GC–MS analysis identified linoleic acid, oleic acid, ethyl palmitate, and ethyl linoleate as the predominant constituents, while carvacrol was detected in lower concentrations. These compounds have previously been associated with membrane disruption, inhibition of microbial growth, attenuation of inflammation, and antioxidant activity, which together may contribute to the beneficial effects observed in PHM-treated fish. However, the present study did not experimentally determine the contribution of individual phytochemicals or evaluate potential synergistic interactions among herbal constituents. Likewise, quantitative phytochemical analyses, HPLC profiling, and bioactivity-guided fractionation were not performed, and molecular pathways such as bacterial efflux pump inhibition, inflammatory cytokine modulation, and erythropoietic regulation were not investigated. Consequently, these proposed mechanisms remain hypothetical and require confirmation through targeted mechanistic studies.

Unlike PHM, garlic supplementation failed to protect fish against *A. veronii* infection, with complete mortality occurring in all garlic-treated groups. Although garlic has been widely reported to possess antimicrobial properties, its efficacy can vary considerably depending on the stability of bioactive organosulfur compounds such as *allicin*, feed processing methods, bacterial strain characteristics, dosage, and resistance mechanisms including biofilm formation and active efflux systems (8, 9, 14). Therefore, the lack of therapeutic efficacy observed in the present study should not be interpreted as evidence that garlic lacks antibacterial activity, but rather as reflecting the specific interaction between the garlic formulation, the multidrug-resistant *A. veronii* isolate, infection intensity, and the experimental conditions employed. These findings emphasize the importance of optimizing herbal formulations before their application as antimicrobial feed additives.

The high cumulative mortality observed in untreated infected fish confirmed the pathogenicity of the isolated *A. veronii* strain and was accompanied by severe clinical signs, extensive gill destruction, and systemic hemorrhage, consistent with previous reports of motile Aeromonas septicaemia (2, 32, 33). In contrast, PHM supplementation reduced mortality to approximately 5.6%, corresponding to nearly 94% survival, and was associated with improved survival compared with oxytetracycline under the conditions of this study. Infection also caused significant reductions in erythrocyte count, hemoglobin concentration, and haematocrit values, indicating anemia and impaired oxygen transport, whereas PHM substantially restored these parameters, suggesting improved physiological status. Similarly, PHM reversed infection-induced neutrophilia and lymphocytopenia, indicating improved regulation of host immune responses during bacterial challenge. Nevertheless, functional immune biomarkers, including lysozyme activity, complement activity, cytokine production, and immune-related gene expression, were not assessed; therefore, the precise immunomodulatory mechanisms responsible for these improvements remain to be determined.

The significant elevation of ALT and ALP activities following infection demonstrated marked hepatic injury and metabolic stress, findings that are consistent with previous studies of *A. veronii* infection in fish. Dietary PHM supplementation significantly reduced liver enzyme activities, suggesting hepatoprotective effects that may be related to the anti-inflammatory and antioxidant-associated properties of its phytochemicals. Likewise, histopathological examination demonstrated that PHM markedly reduced epithelial hyperplasia, lamellar fusion, inflammatory infiltration, necrosis, and degeneration of primary and secondary gill filaments, indicating substantial preservation of tissue architecture. Although these findings strongly support a protective effect of PHM, bacterial burden, inflammatory cytokines, oxidative stress biomarkers, quantitative morphometric analyses, and bacterial biofilm formation were not evaluated, limiting detailed mechanistic interpretation of tissue recovery.

Overall, the present findings demonstrate that dietary PHM supplementation substantially improved survival, physiological status, liver function, and gill tissue integrity in common carp experimentally challenged with *A. veronii*. The observed benefits are likely attributable to the combined antibacterial and host-protective properties of the herbal formulation rather than a single active constituent. From a practical perspective, PHM represents a promising complementary strategy for reducing antibiotic use and supporting sustainable aquaculture. However, before commercial implementation can be recommended, future studies should validate these findings through standardized phytochemical characterization, molecular and immunological investigations, quantitative antimicrobial analyses, Kaplan–Meier survival analysis, safety evaluation in healthy fish, residue assessment, and large-scale field trials under commercial farming conditions.

### 4.2 Study limitations

Although the present study demonstrates the promising protective effects of the polyherbal mixture (PHM) against *Aeromonas veronii* infection in common carp, several limitations should be considered when interpreting the findings. Antibacterial activity was assessed primarily using disc diffusion assays; therefore, quantitative antimicrobial evaluations including minimum inhibitory concentration (MIC), minimum bactericidal concentration (MBC), pharmacodynamic analyses, and time-kill kinetic assays were not performed. Consequently, the antimicrobial potency and the bacteriostatic or bactericidal nature of PHM remain to be established.

The mechanisms underlying the observed protective effects also require further clarification. Although GC–MS identified several bioactive constituents, quantitative phytochemical analyses, HPLC profiling, and bioactivity-guided fractionation were not conducted. Likewise, functional immune biomarkers, oxidative stress parameters, inflammatory cytokines, bacterial burden, biofilm formation, and quantitative histopathological analyses were not evaluated, limiting mechanistic interpretation of the observed improvements.

In addition, the study design did not include non-infected fish receiving PHM supplementation, preventing assessment of the long-term physiological effects and safety of the herbal formulation under normal rearing conditions. Survival outcomes were presented as cumulative mortality percentages rather than Kaplan–Meier survival analyses, and the experimental findings have not yet been validated under commercial farming conditions.

Future studies should therefore combine detailed phytochemical characterization with molecular, microbiological, immunological, and physiological investigations, while incorporating survival modeling, large-scale field trials, and economic evaluations to determine the practical applicability of PHM as a sustainable alternative or complementary strategy to conventional antibiotics in aquaculture.

## 5 Conclusion

The present study demonstrated that *Aeromonas veronii* infection causes severe pathological, hematological, and physiological disturbances in common carp, resulting in high mortality under experimental conditions. Dietary supplementation with the polyherbal mixture (PHM), particularly at the 7.5% inclusion level, significantly enhanced survival, improved hematological parameters and immune-associated responses, reduced alterations in liver enzyme activities, and alleviated gill histopathological damage in infected fish.

PHM-treated fish exhibited substantial protection against *A. veronii* infection, with survival reaching approximately 94% under the experimental conditions employed. In contrast, garlic supplementation alone failed to provide effective protection against the bacterial challenge. The observed protective effects of PHM may be associated with bioactive phytochemical constituents identified through GC–MS analysis, including compounds with reported antimicrobial, antioxidant, and anti-inflammatory properties. However, the precise mechanisms of action and the contribution of individual compounds remain unclear and require further investigation.

These findings indicate that dietary PHM supplementation enhances resistance to *A. veronii* infection and improves several health-related parameters in experimentally challenged fish. Nevertheless, dedicated safety studies incorporating non-infected herb-supplemented control groups are required to fully evaluate the long-term safety, tolerability, and physiological effects of PHM under normal aquaculture conditions.

Future studies should incorporate comprehensive phytochemical characterization, including quantification of phenolics, flavonoids, tannins, and antioxidant activities, together with oxidative stress biomarkers, immune-related indicators, bacterial burden assessments, biofilm inhibition assays, and mechanistic investigations. In addition, large-scale field trials evaluating feed stability, production costs, and practical implementation under commercial farming conditions will be necessary to determine the feasibility of PHM as a complementary strategy for reducing reliance on antibiotics in aquaculture.

## Data Availability

The datasets presented in this study can be found in online repositories. The names of the repository/repositories and accession number(s) can be found in the article/Supplementary material.
