# Supplementary material for: Polyherbal therapy modulates survival, hematological parameters, and gill health in Aeromonas veronii-infected Cyprinus carpio
Source: Front Vet Sci. 2026 Aug 19;13:1889901. doi: 10.3389/fvets.2026.1889901 (PMC13536731; doi:10.3389/fvets.2026.1889901)
Supplement: Supplementary file 1 [file Table_1.docx]

**Supplementary Materials**

**Polyherbal Therapy Modulates Survival, Haematological Parameters, and Gill Health in *Aeromonas veronii*-Infected** ***Cyprinus carpio***

**Authors:**

*Omed I. Abid^1,3,*^, and Nasreen M. Abdulrahman^2^*

**Affiliations:**

¹Sulaimani Veterinary Laboratory, Sulaimani Directorate, Sulaymaniyah ^2^College of Veterinary Medicine, University of Sulaimani, Sulaymaniyah, Iraq ^3^Masi Altuni Company, Sulaymaniyah, Iraq.

***Corresponding Author:** Dr Omed Abid **Email**: [omedgoldfish@gmail.com](mailto:omedgoldfish@gmail.com) **Phone**: +964 770 223 7826

**ORCID IDs:** 0000-0002-6415-9163

**Running title:** Polyherbal Protection Against *A. veronii* Infection in Carp

Supplenetary Table S 1: Recording water parameter

| Water Quality Parameter | | | | | | | | | |
| --- | --- | --- | --- | --- | --- | --- | --- | --- | --- |
| Treatments | 1 Days | | | 7 Days | | | 14 Days | | |
|  | DO. | Temp. | PH. | DO. | Temp. | PH. | DO | Temp. | PH. |
| Control | 5.77 | 28.2 | 7.72 | 5.42 | 28.2 | 7.72 | 5.32 | 28.1 | 7.78 |
| Infected | 5.76 | 28.2 | 7.97 | 6.32 | 28.3 | 7.19 | - | - | - |
| OTC feed | 6.09 | 28 | 7.71 | 6.16 | 27.9 | 7.58 | 5.28 | 28.2 | 7.72 |
| OTC bath | 6.7 | 26.7 | 6.70 | 5.47 | 27.6 | 6.66 | 5.47 | 28.3 | 7.35 |
| Garlic 2.5 | 5.65 | 27 | 6.91 | 5.92 | 26.8 | 7.72 | - | - | - |
| Garlic 5 | 5.40 | 26.9 | 7.16 | 6.38 | 27.6 | 7.81 | - | - | - |
| Garlic 7.5 | 6.38 | 27.6 | 7.75 | 6.39 | 26.7 | 7.72 | - | - | - |
| Pom 2.5 | 6.19 | 26.9 | 7.57 | 6.25 | 7.4 | 7.58 | 6.14 | 27.6 | 7.72 |
| Pom 5 | 5.37 | 27.4 | 7.48 | 5.67 | 27.9 | 7.72 | 6.05 | 27.4 | 7.49 |
| Pom 7.5 | 5.29 | 27.9 | 7.52 | 5.31 | 27.9 | 7.52 | 5.46 | 27.4 | 7.62 |
| PHM 2.5 | 5.63 | 27.2 | 7.54 | 5.70 | 28.2 | 7.56 | 6.16 | 26.7 | 7.81 |
| PHM 5 | 5.81 | 27.7 | 7.51 | 5.60 | 27.7 | 7.56 | 5.70 | 26.9 | 7.59 |
| PHM 7.5 | 4.70 | 27.5 | 7.4 | 5.18 | 28 | 7.38 | 6.17 | 27.3 | 7.59 |

**Supplenetary Figurs S 1- 21**

Suppl. Fig 1: Light microscopic structures of infected gill on day 8 exhibited: a and b: Severe degree of primary lamellar degeneration with epithelial hyperplasia, vasodilation (CV), and marked heterophil infiltration (red arrows). c and d: severe hyperplasia of chloride (yellow arrows) and mucus cells (black arrows); PI: Primary lamella, SI: Secondary lamella, EP: Epithelial hyperplasia (H&E stain).

Suppl. Fig 2: Light microscopic structures of infected gill on day 14 showed: a-c: Marked epithelial hyperplasia and severe congestion in the primary lamella, with moderate infiltration of inflammatory cells (red arrows), marked primary lamellar degeneration, marked chloride hyperplasia (yellow arrows), and mucus hyperplasia (black arrows); PI: Primary lamella, SI: Secondary lamella, EP: Epithelial hyperplasia (H&E stain).

Suppl. Fig 3: Light microscopic structures of treated gill with OCT added in feed on day 8 displayed: a and b: Moderate degeneration of primary and secondary filaments, moderate epithelial hyperplasia, and severe interstitial edema in secondary filaments (E) with congestion (CV). c and d: Moderate mucus hyperplasia (black arrows) and mild inflammatory reaction (red arrows); PI: Primary lamella, SI: Secondary lamella, EP: Epithelial hyperplasia (H&E stain).

Suppl. Fig 4: Light microscopic structures of treated gill with OCT added in feed on day 14 presented: a-c: Marked degeneration of primary and secondary filaments, severe epithelial hyperplasia, and severe interstitial edema in secondary filaments (E) with congestion (CV). d: Mild chloride hyperplasia (yellow arrows) and moderate inflammatory reaction (red arrows); PI: Primary lamella, SI: Secondary lamella, EP: Epithelial hyperplasia (H&E stain).

Suppl. Fig 5: Light microscopic structures of treated gill with OCT by bath on day 8 exhibited: a-c: Marked degeneration of primary and secondary filaments with lifting, moderate epithelial hyperplasia, moderate chloride hyperplasia (yellow arrows), and mild inflammatory reaction (red arrows); PI: Primary lamella, SI: Secondary lamella, EP: Epithelial hyperplasia (H&E stain).

Suppl. Fig 6: Light microscopic structures of treated gill with OCT by bath on day 14 presented: a-c: Severe degeneration of primary and secondary filaments with clubbing (CL), mild-moderate epithelial hyperplasia, mild chloride hyperplasia (yellow arrows) and mild inflammatory reaction (red arrows); PI: Primary lamella, SI: Secondary lamella, EP: Epithelial hyperplasia (H&E stain).

Suppl. Fig 7: Light microscopic structures of treated gill with 2.5 gm/kg/bw of garlic on day 8 exhibited: a and b: Marked degeneration of primary and secondary filaments with lifting and clubbing (CL), moderate epithelial hyperplasia, and moderate interstitial odema in secondary filaments (E) with congestion (CV). c and d: Moderate chloride hyperplasia (yellow arrows) and mild-moderate inflammatory reaction (red arrows); PI: Primary lamella, SI: Secondary lamella, EP: Epithelial hyperplasia (H&E stain).

Suppl. Fig 8: Light microscopic structures of treated gill with 5 gm/kg/bw of garlic on day 8 exhibited: a-c: Marked degeneration of primary and secondary filaments, marked epithelial hyperplasia, severe chloride and mucus hyperplasia (yellow and black arrows) and moderate inflammatory reaction (red arrows); PI: Primary lamella, SI: Secondary lamella, EP: Epithelial hyperplasia (H&E stain).

Suppl. Fig 9: Light microscopic structures of treated gill with 7.5 gm/kg/bw of garlic on day 8 exposed: a-c: Severe vacuolation of primary and secondary filaments, severe epithelial hyperplasia, marked odema (E) with vascular congestion (CV), severe chloride and mucus hyperplasia (yellow and black arrows) and moderate inflammatory reaction (red arrows); PI: Primary lamella, SI: Secondary lamella, EP: Epithelial hyperplasia (H&E stain).

Suppl. Fig 10: Light microscopic structures of treated gill with 2.5 gm/kg/bw of Pomegranate on day 8 presented: a and b: Moderate vacuolation of primary and secondary filaments with clubbing of secondary one (CL), moderate epithelial hyperplasia and severe vascular congestion (CV), moderate chloride hyperplasia (yellow arrows) and mild inflammatory reaction (red arrows); PI: Primary lamella, SI: Secondary lamella, EP: Epithelial hyperplasia (H&E stain).

Suppl. Fig 11: Light microscopic structures of treated gill with 5 gm/kg/bw of Pomegranate on day 8 revealed: a-c: Moderate degeneration of primary and secondary filaments, mild-moderate epithelial and chloride hyperplasia (yellow arrows), and mild inflammatory reaction (red arrows); PI: Primary lamella, SI: Secondary lamella, EP: Epithelial hyperplasia (H&E stain).

Suppl. Fig 12: Light microscopic structures of treated gill with 7.5 gm/kg/bw of Pomegranate on day 8 revealed: a-c: Moderate degeneration of primary and secondary filaments, mild odema (E), mild-moderate epithelial hyperplasia, and mild inflammatory reaction (red arrows); PI: Primary lamella, SI: Secondary lamella, EP: Epithelial hyperplasia (H&E stain).

Suppl. Fig 13: Light microscopic structures of treated gill with 2.5 gm/kg/bw of Pomegranate on day 14 displayed: a-c: Moderate degeneration of primary and secondary filaments with fusion of secondary one (Fu), mild-moderate epithelial hyperplasia, mild chloride hyperplasia (yellow arrows) and mild inflammatory reaction (red arrows); PI: Primary lamella, SI: Secondary lamella, EP: Epithelial hyperplasia (H&E stain).

Suppl. Fig 14: Light microscopic structures of treated gill with 5 gm/kg/bw of Pomegranate on day 14 revealed: a and b: Mild-moderate degeneration of primary and secondary filaments with vasodilation (CV), mild-moderate epithelial and mild chloride hyperplasia (yellow arrows), and mild inflammatory reaction (red arrows); PI: Primary lamella, SI: Secondary lamella, EP: Epithelial hyperplasia (H&E stain).

Suppl. Fig 15: Light microscopic structures of treated gill with 7.5 gm/kg/bw of Pomegranate on day 14 revealed: a-c: Mild-moderate vacuolation of primary and secondary filaments, mild-moderate epithelial and chloride (yellow arrows) hyperplasia, mild mucus hyperplasia (black arrows) and mild inflammatory reaction (red arrows); PI: Primary lamella, SI: Secondary lamella, EP: Epithelial hyperplasia (H&E stain).

Suppl. Fig 16: Light microscopic structures of treated gill with 2.5 gm/kg/bw of poly herb on day 8 revealed: a-c: Mild-moderate vacuolation of primary and fusion (Fu) of secondary filaments, mild-moderate epithelial and chloride (yellow arrows) hyperplasia, and mild inflammatory reaction (red arrows); PI: Primary lamella, SI: Secondary lamella, EP: Epithelial hyperplasia (H&E stain).

Suppl. Fig 17: Light microscopic structures of treated gill with 5 gm/kg/bw of poly herb on day 8 exhibited: a and b: Mild-moderate vacuolation of primary and fusion (Fu) of secondary filaments, mild epithelial and chloride (yellow arrows) hyperplasia; PI: Primary lamella, SI: Secondary lamella, EP: Epithelial hyperplasia (H&E stain).

Suppl. Fig 18: Light microscopic structures of treated gill with 7.5 gm/kg/bw of poly herb on day 8 showed: a and b: Mild-moderate vacuolation of primary and sloughing of secondary filaments, mild epithelial and chloride (yellow arrows) hyperplasia; PI: Primary lamella, SI: Secondary lamella, EP: Epithelial hyperplasia (H&E stain).

Suppl. Fig 19: Light microscopic structures of treated gill with 2.5 gm/kg/bw of poly herb on day 14 presented: a and b: Mild vacuolation of primary and clubbing (CL) of secondary filaments, mild-moderate epithelial and mild chloride (yellow arrows) hyperplasia; PI: Primary lamella, SI: Secondary lamella, EP: Epithelial hyperplasia (H&E stain).

Suppl. Fig 20: Light microscopic structures of treated gill with 5 gm/kg/bw of poly herb on day 14 exhibited: a and b: Mild-moderate vacuolation of primary and secondary filaments, mild epithelial and chloride (yellow arrows) hyperplasia,; PI: Primary lamella, SI: Secondary lamella, EP: Epithelial hyperplasia (H&E stain).

Suppl. Fig 21: Light microscopic structures of treated gill with 7.5 gm/kg/bw of poly herb on day 14 displayed: a and b: Mild-moderate degeneration of primary filaments, mild sloughing of secondary filaments, mild chloride (yellow arrows) hyperplasia; PI: Primary lamella, SI: Secondary lamella, EP: Epithelial hyperplasia (H&E stain).
